# Essential Role of TEA Domain Transcription Factors in the Negative Regulation of the MYH 7 Gene by Thyroid Hormone and Its Receptors

**DOI:** 10.1371/journal.pone.0088610

**Published:** 2014-04-29

**Authors:** Hiroyuki Iwaki, Shigekazu Sasaki, Akio Matsushita, Kenji Ohba, Hideyuki Matsunaga, Hiroko Misawa, Yutaka Oki, Keiko Ishizuka, Hirotoshi Nakamura, Takafumi Suda

**Affiliations:** 1 Second Division, Department of Internal Medicine, Hamamatsu University School of Medicine, Hamamatsu, Shizuoka, Japan; 2 Department of Laboratory Medicine, Hamamatsu University School of Medicine, Hamamatsu, Shizuoka, Japan; 3 Department of Internal Medicine, Kuma Hospital, Kobe, Hyogo, Japan; Ecole Normale Supérieure de Lyon, France

## Abstract

MYH7 (also referred to as cardiac myosin heavy chain β) gene expression is known to be repressed by thyroid hormone (T3). However, the molecular mechanism by which T3 inhibits the transcription of its target genes (negative regulation) remains to be clarified, whereas those of transcriptional activation by T3 (positive regulation) have been elucidated in detail. Two MCAT (muscle C, A, and T) sites and an A/T-rich region in the MYH7 gene have been shown to play a critical role in the expression of this gene and are known to be recognized by the TEAD/TEF family of transcription factors (TEADs). Using a reconstitution system with CV-1 cells, which has been utilized in the analysis of positive as well as negative regulation, we demonstrate that both T3 receptor (TR) β1 and α1 inhibit TEAD-dependent activation of the MYH7 promoter in a T3 dose-dependent manner. TRβ1 bound with GC-1, a TRβ-selective T3 analog, also repressed TEAD-induced activity. Although T3-dependent inhibition required the DNA-binding domain (DBD) of TRβ1, it remained after the putative negative T3-responsive elements were mutated. A co-immunoprecipitation study demonstrated the *in vivo* association of TRβ1 with TEAD-1, and the interaction surfaces were mapped to the DBD of the TRβ1 and TEA domains of TEAD-1, both of which are highly conserved among TRs and TEADs, respectively. The importance of TEADs in MYH7 expression was also validated with RNA interference using rat embryonic cardiomyocyte H9c2 cells. These results indicate that T3-bound TRs interfere with transactivation by TEADs *via* protein-protein interactions, resulting in the negative regulation of MYH7 promoter activity.

## Introduction

Cardiac muscle contraction in vertebrates is mediated by two molecular motors, MYH6 and MYH7, which are also referred to as myosin heavy chain α and β, respectively [1], [2]. Actin-induced ATPase activity of the MYH7 protein is two- to three-fold less than that of the MYH6 protein, and a relatively subtle change in the MYH6/MYH7 ratio has been shown to affect cardiac function. For example, myocyte fragments containing 12% MYH6 protein develop 52% greater power output than that by myocyte fragments without MYH6 [3]. A decrease in MYH6 expression and increase in MYH7 expression is observed in rodent models with experimental heart failure [4] and myocardial infarction [5]. Thus, the transition from MYH6 to MYH7 found in heart failure is thought to be a maladaptive response [6]. The thyroid hormone (T3) signal is shown to strongly activate transcription of the MYH6 gene (positive regulation) and reduces that of the MYH7 gene (negative regulation) in mice and rats [1], [2], [7]. MYH7 represents 90% of total cardiac myosin heavy chains in rodent embryos, with the remainder being MYH6. However, the MYH6/MYH7 ratio is markedly inversed by an elevation in serum T3 and T4 levels after birth. Interestingly, a T3-induced decrease in MYH7 and increase in MYH6 levels are similar to their gene expression profiles found in physiological cardiac hypertrophy induced by exercise [6], [8], while cardiac function has been shown to be reduced in hypothyroidism. Overexpression of the T3 receptor (TR) isoforms [9] or type-2 deiodinase [10] in the mouse heart is known to improve cardiac function. However, abnormally high concentrations of serum thyroid hormone may induce tachycardia and/or cardiac ischemia [6]. Thus, developing a T3 analog that does not have a deleterious effect on the heart may be a promising treatment for heart failure.

TRs belong to the nuclear hormone receptor (NHR) superfamily, and are encoded by two separate alleles, TRα and β. The TRα gene generates TRα1, Δα1, α2, Δα2, and α3 through alternative splicing while the TRβ gene generates TRβ1, β2, β3, and Δβ3 with the use of the different promoters. Among them, TRα1, β1 and β2 are regarded as the major functional TRs [11]. In contrast to the limited distribution of the TRβ2 protein (pituitary, hypothalamus, inner ear, and retina), TRα1 and TRβ1 are ubiquitously expressed [11]. Although various conditions including T3 administration [12], [13] and pressure overload [4] influence the expression levels of endogenous TRα1 and TRβ1, the amount of TRα1 mRNA in the stable condition of cardiac tissue was shown to be two- to three-fold higher than that of TRβ1 mRNA [4], [7]. Thus, TRα1 is regarded as the major mediator of T3 signaling for cardiac function [14]. The molecular mechanism of transcriptional activation by T3 (positive regulation) has been clarified in detail [11]. In positively regulated genes, including the MYH6 gene [7], [15], TR heterodimerizes with the retinoid X receptor and each receptor directly recognizes one of the half-sites (typically, AGGTCA) of T3-responsive element (TRE) via their DNA-binding domains (DBDs) [11]. Unliganded TR on TRE associates with co-repressors, such as nuclear receptor co-repressor (NCoR) or silencing mediator for retinoid and thyroid hormone receptors (SMRT), resulting in the active suppression of target genes (silencing) [11], [16]. Upon T3 binding to TR *via* its ligand-binding domain (LBD), silencing is relieved by the dissociation of NCoR or SMRT from TR. T3-bound TR (T3/TR) subsequently recruits co-activators including the p160 family, cAMP response element-binding protein-binding protein (CBP)/p300, and MED1, all of which interact with T3/TR *via* their LXXLL motifs (L is leucine and X is any amino acid) and activate the transcription of target genes (transactivation). The p160 family and CBP/p300 exhibit intrinsic histone acetyltransferase (HAT) activity while MED1 is a constituent of the Mediator complex that precisely mediates information of liganded NHRs and DNA-binding transcription factors to RNA polymerase II [17].

In contrast to positive regulation by liganded NHR, little is known about the molecular mechanism of negative regulation by T3, and a strategy to investigate it has not yet been established [16], [18]. Previous studies reported that TRβ1, but not TRα1, mediates negative regulation of the MYH7 gene [4], [19]. However, this should be reconsidered because TRβ1-deficient mice exhibited the inhibition of MYH7 expression by T3 presumably *via* the remaining TRα1 [20]. Although the existence of a negative TRE (nTRE) has been postulated in the MYH7 promoter as sequences homologous to a single half-site [2], [21], its function has not been experimentally verified. The best example of T3-dependent negative regulation was described for the gene encoding the β subunit of thyrotropin (thyroid stimulating hormone β subunit, TSHβ) [16], [22]. Because TSHβ expression in the pituitary thyrotroph increases in hypothyroidism, one would presume that unliganded TR activates the TSHβ gene. Moreover, it has been proposed that unliganded TR may be critical for the global activity of the TSHβ promoter. Based on this, a short DNA sequence (GGGTCA) similar to a single half-site was reported as nTRE [22], which is required for transactivation of the TSHβ promoter by unliganded TR in HEK293 cells [23]. Nevertheless, TSHβ expression was not decreased, but rather increased in mice completely deficient in both TRβ and TRα genes {[16] and references therein}. This finding implies that unliganded TR *per se* is not a transcriptional activator for the TSHβ gene and raises the question about the definition of nTRE [24]. We previously demonstrated that reported nTRE [23] is dispensable for inhibition of the TSHβ gene by T3/TR using monkey kidney-derived CV-1 cells [25] co-expressed with two transcription factors, GATA2 and Pit1, which are essential for TSHβ gene activity and are critical determinants of thyrotroph development [16], [24], [26], [27]. We proposed the tethering model, in which the DBD of TR does not directly recognize DNA, but associates with the GATA2-Zn finger domain *via* protein-protein interactions [16], [27]. In this scenario, TR interferes with the transactivating function of GATA2 in a T3-dependent manner, resulting in the TSHβ gene repression.

As in the case of the TSHβ gene, MYH7 expression was also maintained in mice completely deficient of both the TRβ and TRα genes [20]. Thus, MYH7 expression is thought to be maintained by a factor other than unliganded TRs. Morkin et al. [1] analyzed the 5′ flanking region (nt −1285 to nt +126) of the human MYH7 gene using a primary culture of rat fetus cardiomyocytes. The results of this study showed that the short sequence encompassing nt −277 to −298 is critical for MYH7 expression. Similar conclusions were made for the rat and rabbit MYH7 genes [28], [29]. These studies suggested that two MCAT (muscle C, A, and T) sites and an A/T-rich sequence in this region play a pivotal role in basal expression of the MYH7 gene. The importance of these elements was also confirmed by a study with transgenic mice in which the reporter gene harboring 5.6 kb of the mouse MYH7 promoter was introduced [30]. Promoter analyses with rat cardiomyocytes and skeletal muscle-derived cells have suggested that TEAD/TEF family transcription factors (referred to as TEADs) recognize MCAT sequences and the A/T-rich element in the MYH7 gene [1], [2], [31], [32] *via* the TEA domain, which is highly conserved among TEADs [31]–[34]. The TEAD family consists of four members, i.e., TEAD-1 (TEF-1/NTEF), TEAD-2 (ETF/ETEF-1/TEF-4), TEAD-3 (DTEF-1/TEF-5ETFR-1), and TEAD-4 (RTEF-1/TEF-R1/TEF-3/ETFR-2/FR-191) [32]. While TEAD-2 is expressed temporarily in the developmental stage, the expression of other TEADs has been detected in adult tissues [32]. Although TEAD-1 knockout mice show embryonic lethality, mice in which TEAD-1 was conditionally overexpressed in cardiomyocytes exhibited heart failure with increased MYH7 gene expression [35]. In contrast to the ubiquitous localization of TEAD-1, TEAD-3 expression was shown to be more abundant in the heart than in skeletal muscle [32], [33] and TEAD-4 was mainly expressed in skeletal muscle and the lung [33]. Maeda et al. [36] suggested that TEAD-3 mediates α1-adrenaline signals, which play a pivotal role in pathological cardiac hypertrophy accompanied with increases in the expression of MYH7 [2], [32], [37].

Interestingly, *in vivo* [21], [38] and *in vitro* [15], [39] studies have suggested that negative regulation by T3/TR can be detected in the rodent MYH7 promoter containing these MCAT sites and an A/T-rich region. It may be reasonable that T3-dependent repression is detectable only when T3/TR interferes with the activity of DNA-binding transcription factors, which maintain overall transcription levels [16], [18]. Based on this hypothesis, we explored the molecular mechanism by which T3/TR regulates TEAD-dependent activity of the MYH7 promoter. Because endogenous TRα1 and TRβ1 expression levels were shown to be markedly affected by various conditions including T3 administration [12], [13] and cardiac pressure overload [4], we used a cell culture system. In addition to rat neonatal cardiac muscle-derived H9c2 cells [40], we employed kidney-derived CV-1 cells [25] because they have often been used not only in studies of the MYH6 gene (positive regulation) [15], but also of the TSHβ gene (negative regulation) [16], [26], [27]. Our reconstitution system revealed that unliganded TR is not the transcriptional activator for the MYH7 gene, and that putative nTREs [2], [21] are dispensable for T3-induced inhibition. We propose here a tethering mechanism in which T3/TR inhibits the transcriptional activity of TEADs *via* the interaction of TR-DBD with the TEA domain of TEADs, which causes the inhibition of MYH7 promoter activity. Although distinct transcription factors, i.e., TEADs and GATA2, maintain the basal activities of MYH7 and TSHβ genes, respectively, the tethering mechanism is thought to be a common aspect in the T3-dependent negative regulation of these two genes.

## Materials and Methods

### Plasmid constructions

Because the firefly luciferase-based reporter gene may be artificially suppressed by T3/TR [16], [18], [26], we employed chloramphenicol acetyltransferase (CAT)- and modified *Renilla* luciferase (hRluc)-based reporter systems (Promega Corp., Madison, WI, USA). The human MYH7 promoter encompassing nt −293/+125 was fused with the CAT reporter gene and hRluc reporter gene, generating MYH7-CAT and MYH7-hRluc, respectively. Del-1-CAT (deletion of distal MCAT and the A/T-rich region) and Del-2-CAT (deletion of the A/T-rich region and both MCATs) were generated using PCR amplification of −254/+125 and −198/+125 of the human MYH7 promoter, respectively. Using site-directed mutagenesis (Stratagene, La Jolla, CA, USA), we mutated distal MCAT (Mut-1 and Mut-dM), the A/T-rich region (Mut-2 and Mut-AT), distal MCAT plus the A/T-rich region (Mut-3), proximal MCAT (Mut-4 and Mut-pM), both MCATs plus the A/T-rich region (Mut-5 and Mut-MAM) and all of the reported nTREs (MYH7-mnTRE-CAT). Expression plasmids for human TRβ1 (pCMX-hTRβ1), human TRα1 (pCMX-hTRα1), and the deletion constructs of hTRβ1 (C1 and C2), and GST-DBD have been described previously [16], [27]. The expression plasmid (pcDNA3-6myc-TEAD-1) for the N-terminal six tandem myc-tagged mouse TEAD-1 (6myc-TEAD-1) was a gift from Dr. Michinori Kitagawa (Kumamoto University, Kumamoto, Japan). Expression plasmids for mouse TEAD-3/DTEF-1 (pXJ40-DTEF-1) and mouse TEAD-4/RTEF-1 (pXJ40-RTEF-1) were provided by Dr. Tomoji Maeda (Iwate Medical University, Morioka, Japan). Using site-directed mutagenesis, a stop codon was introduced at codon 220 in pcDNA3-6myc-TEAD-1 to generate TEAD-1ΔC. The DNA fragment encompassing codons 101–426 of mouse TEAD-1 was amplified by PCR and subcloned into the Not1/Xho1 site in the pcDNA3-6myc vector to generate pcDNA3-6myc-TEDA-1-TEA. The TEA domain of mouse TEAD-1 (codons 1–101) was amplified by PCR and was subcloned into the BamH1/Xho1 site in the pGEX-4T-1 vector (Amersham Biosciences, Piscataway, NJ, USA) to generate pGEX-4T-1-TEA (GST-TEA). All mutated sequences and subcloning sites were confirmed by sequencing.

### Cell culture and transient transfection

Monkey kidney-derived CV-1 cells [25] and rat neonatal cardiac muscle-derived H9c2 cells [40] were grown in a monolayer culture at 37°C under CO_2_/air (1∶19) in Dulbecco's modified Eagle's medium (DMEM) containing 10% (v/v) fetal calf serum (FCS), penicillin G (100 units/ml), and streptomycin (100 µg/ml). CV1 cells were trypsinized and plated in 60 mm dishes for 24 h prior to transient transfection using the calcium-phosphate technique. Cells were transfected at a density of 2×10^5^ cells per well with 2.0 µg of the MYH7-CAT reporter gene, 0.4 µg of expression vectors for the TEAD family transcription factors and/or TRs, and 1.8 µg of the β-galactosidase expression vector, pCH111 (a modified version of pCH110, Pharmacia LKB Biotechnology, Piscataway, NJ, USA). The total amount of the expression plasmid was adjusted with the pCMX empty vector (7.2 µg of DNA in total per dish). After cells were exposed to calcium phosphate/DNA precipitates for 20 h, the medium was replaced with fresh DMEM containing 10% FCS depleted of thyroid hormone [26], [41] or medium supplemented with T3. Cells were harvested after incubation for an additional 24 h, and CAT activity was measured as described previously [27]. H9c2 cells at a density of 2×10^5^ cells per well were transfected with 4.0 µg of the MYH7-hRluc reporter gene and 0.4 µg of expression vectors for TRβ1 using Lipofectamine reagent (Life Technologies, Carlsbad, CA, USA) according to the manufacturer's protocol. After 5 h incubation, the medium was replaced with fresh DMEM containing 5% FCS depleted of thyroid hormones or medium supplemented with T3. *Renilla* luciferase activities were measured with the *Renilla* Luciferase Assay System (Promega Corp.) using a Lumicounter 700 (Microtech Nichi-on, Chiba, Japan). CAT and *Renilla* luciferase activities were normalized for transfection efficiency determined by the β-galactosidase assay [27]. We performed transfections with pCMV-CAT (5.0 ng/well) or pGL4.74[hRLuc/TK] (2.0 µg/well), for each reporter assay, the magnitudes of which were adjusted to a value of 100.

### Gel shift assay

The PG1-probe (wild-type the distal MCAT site and an A/T-rich element, sense; 5′-gctgtggaatgtgaggcctggcctgggagatatttttgctgc-3′ and antisense; 5′-gcagcaaaaatatctcccaggccaggcctcacattccacagc-3′), PG2-probe (wild-type A/T-rich element, sense; 5′-ggcctgggagatatttttgctgcact-3′ and antisense; 5′-agtgcagcaaaaatatctcccaggcc-3′), and PG3-probe (wild-type proximal MCAT, sense; 5′-gcacagtccatgccataaca-3′ and antisense; 5′-tgttatggcatggactgtgc-3′) were labeled with γ-^32^P-ATP using T4 polynucleotide kinase (Toyobo, Tokyo, Japan). The 6myc-TEAD-1 protein was produced with an *in vitro* translation kit (Promega Corp.). γ-^32^P-labeled probes and TEAD-1 proteins were incubated for 30 min on ice in 20 µl binding buffer containing 10 mM Tris-HCl (pH 7.6), 50 mM KCl, 0.05 mM EDTA, 2.5 mM MgCl_2_, 8.5% glycerol, 1 mM dithiothreitol, 0.5 µg/ml poly (dI-dC), 0.1% Triton X-100, and 1 mg/ml nonfat dry milk. A 100-fold molar excess of cold oligonucleotides for PG1, PG2, PG3, M1 (mutation of the A/T rich region, sense; 5′-gctgtggaatgtgaggcctggcctgggagatgggtttgctgc-3′ and antisense; 5′-gcagcaaacccatctcccaggvvaggcctcacattccacagc -3′), M2 (mutation of distal MCAT, sense; 5′-gctgtggacgttgaggcctggcctgggagatatttttgctgc-3′ and antisense; 5′-gcagcaaaaatatctcccaggccaggcctcaacgtccacagc-3′), M3 (mutation of distal MCAT and the A/T rich region, sense; 5′-gctgtggacgttgaggcctggcctgggagatgggtttgctgc-3′ and antisense; 5′- gcagcaaacccatctcccaggccaggcctcaacgtccacagc-3′), M4 (mutation of the A/T rich region, sense; 5′- ggcctgggagatgggtttgctgcact-3′ and antisense; 5′-agtgcagcaaacccatctcccaggcc-3′), M5 (mutation of proximal MCAT, sense; 5′- gcacagtccgggccataaca-3′ and antisense; 5′-tgttatggcccggactgtgc-3′), and non-specific oligo-DNA (NS, sense; 5′-GGTACCAAGCTTGTGGAGATCT-3′ and antisense; 5′-AGATCTCCACAAGCTTGGTACC-3′) were used as cold competitors. DNA–protein complexes were resolved by electrophoresis on a 5% polyacrylamide gel at 100 V for 80 min at room temperature. Anti-c-myc antibody (Santa Cruz Biotechnology Inc., Santa Cruz, CA, USA) was added for the supershift assay. The gel was dried, and labeled bands were visualized using the BAS-1000 autoradiography system (Fuji Film, Tokyo, Japan).

### Immunoprecipitation and Western blotting

Expression vectors for FLAG-tagged TRβ1 (pCMX-FLAG-rTRβ1) and 6myc-TEAD-1 (pcDNA3-6myc-TEAD-1) were co-transfected into CV-1 cells using the calcium phosphate method. After cells had been exposed to calcium phosphate/DNA precipitates for 24 h, the medium was replaced with fresh DMEM containing 5% FCS depleted of thyroid hormone [41] or medium supplemented with T3. After a 24-h incubation in the presence or absence of 1 µM T3, CV-1 cells were harvested and washed twice with ice-cold PBS. Cell pellets were lysed in hypotonic buffer [20 mM HEPES (pH 7.9), 10 mM KCl, 10% glycerol, 1 µM EDTA, 0.2% Nonidet-P40, 3 µg/ml aprotinin, and leupeptin] and incubated on ice for 15 min. After centrifugation at 14,000 rpm for 5 min, the pellet was resuspended in a high-salt buffer [20 mM HEPES (pH 7.9), 420 mM NaCl, 20% glycerol, 1 µM EDTA, 0.2% Nonidet P-40, 3 µg/ml aprotinin, and leupeptin] and gently agitated at 4°C for 30 min. The supernatants were collected after centrifugation at 14,000 rpm for 10 min and incubated with anti-FLAG M2 affinity gel (Sigma, St. Louis, MO, USA) in binding buffer [150 mM NaCl, 20 mM Tris HCl (pH 7.5), 0.3% Nonidet P-40, 3 µg/ml aprotinin, and leupeptin] at 4°C overnight. After four washes with hypotonic buffer, the immunocomplexes were resolved by SDS-PAGE, Western blotted using the anti-c-myc antibody (Santa Cruz Biotechnology Inc. TX, USA), and analyzed by the anti-FLAG M2 antibody (Sigma). To confirm the expression of wild-type and mutant TRs transfected into CV1 cells, a 0–3 µg/6 cm diameter dish of the expression plasmids for wild-type TRβ1, FLAG-tagged TRβ1, C1, C2 and FLAG-tagged TRα1 were transfected and whole-cell extracts were subjected to Western blotting with antibodies against N-terminal region of TRβ1 (PP-H3825A-00, Perseus proteomics, Japan), FLAG (M2, Sigma) and TRα1 (sc-772, Santa Cruz Biotechnology Inc.). To assess the expression level of endogenous TEAD-1, TRα1 and TRβ1 in H9c2 cells, the cells in a 10-cm dish were harvested, and whole cell extracts were fractionated by SDS-PAGE and subjected to Western blotting with the antibodies against TEAD-1 (Becton Dickinson Transduction Laboratories, NJ, USA), N-terminal region of TRβ1 (PP-H3825A-00) and TRα1 (sc-772), respectively.

### GST pull-down assay

*E. coli* (DH5α) that had been transformed with pGEX-4T-1-TEA (GST-TEA) or GST-DBD was induced with 0.1 mM isopropyl-1-thio-β-galactopyranoside for 4 h. The *E. coli* pellet was sonicated, and fusion proteins were mixed with glutathione-Sepharose beads (Amersham Pharmacia Biotech, Uppsala, Sweden) for purification. Receptor proteins (TRβ1, C1 and C2), 6myc-TEAD-1, and TEAD-1 mutants (TEAD-1ΔC and ΔTEA) were translated *in vitro* using rabbit reticulocyte lysate (Promega) in the presence of ^35^S-methionine. Radiolabeled proteins were incubated with GST fusion proteins in binding buffer [150 mM NaCl, 20 mM Tris HCl (pH 7.5), 0.3% Nonidet P-40, 1 mM dithiothreitol, 0.5 mM phenylmethylsulfonyl fluoride, 2 µg/ml leupeptin, and 2 µg/ml aprotinin] for 3 h at 4°C and washed three times with the binding buffer. The bound fraction was analyzed by 10–14% SDS-PAGE and visualized using a BAS-1000 autoradiography system (Fuji Film, Tokyo, Japan).

### RNA interference

Six µl of Lipofectamine 2000 reagent (Life Technologies) plus 300 µl of Opti-MEM-I was mixed with 300 µl of Opti-MEM-I containing 1.5 µl of 50 µM siRNA, Rn Tead1 5, or Rn Tead1 6 (Qiagen, Hilden, Germany). The mixture was incubated at room temperature for 20 minutes and then applied to a monolayer of H9c2 cells cultured in a 6-cm dish. After a 48-h culture, the cells were harvested and used for Western blotting or RT-PCR.

### Real-time reverse transcription polymerase chain reaction (RT-PCR)

H9c2 cells cultured in 10% FCS were incubated, and total RNAs were purified by the acid guanidinium thiocyanate-phenol-chloroform extraction method. One µg total RNA was mixed with random hexanucleotides and 200 units of Moloney murine leukemia virus reverse transcriptase (Invitrogen Corp., Carlsbad, CA, USA) for first-strand cDNA synthesis. Using the SYBR Green I kit and a LightCycler (Roche Diagnostics, Mannheim, Germany), precipitated cDNA was quantified by real-time PCR using the following primers: forward primer (5′-CGAGTCCCAGGTCAACAA-3′) and reverse primer (5′-GGCTTCACAGGCATCCTTA-3′). The cDNA for glyceraldehyde-3-phosphate dehydrogenase (GAPDH) was also amplified with the forward primer (5′-TGAACGGGAAGCTCACTGG-3′) and reverse primer (5′-TCCACCACCCTGTTGGCTGTA-3′). The thermal cycling conditions were 10 min at 95°C, followed by 50 cycles of 10 sec at 95°C for denaturing, 10 sec at 62°C for annealing, and 7 sec at 72°C for extension. PCR signals were analyzed using LightCycler software version 3.5 (Roche Diagnostics).

### Statistical analysis

Each CAT or *Renilla* luciferase reporter assay was performed in duplicate three or more times, and each result was expressed as the mean ± S.D. Significance was examined by ANOVA and Fisher's protected least significant difference test using Stat View 4.0 software (Abacus Concepts, Berkeley, CA, USA). *P*<0.05 was considered significant.

## Results

### TEADs stimulate the MYH7 promoter encompassing two MCAT sites and an A/T-rich element in CV-1 cells

**Figure 1:**
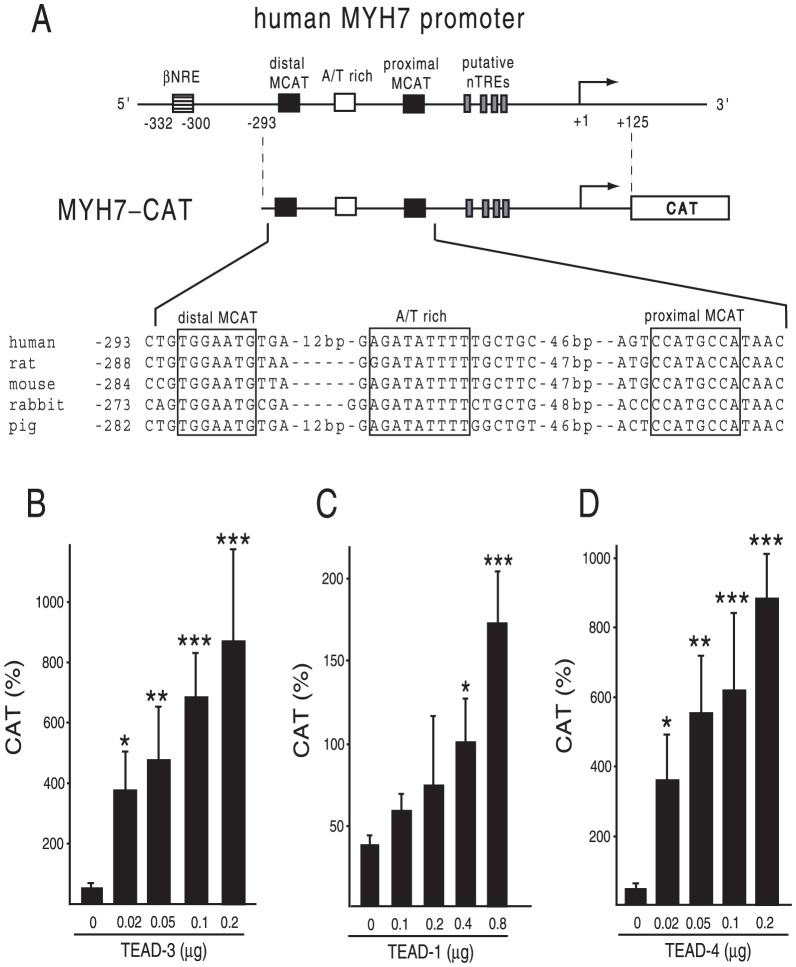
TEAD family transcription factors activate the human MYH7 promoter. (A) Schematics of the human MYH7 promoter region (upper panel) and structure of MYH7-CAT (middle panel). The two reported MCAT sites, an A/T-rich region and four putative negative T3-responsive-elements (nTREs) are indicated as boxes. The transcription start site is indicated as +1. Constitutive repressive sequence (βNRE) at nt −332/−300 is not included in MYH7-CAT. The DNA sequences of two MCAT sites and an A/T-rich region among several species are indicated (lower panel). (B–D) TEAD-3 (B), TEAD-1 (C), and TEAD-4 (D) transactivate MYH7-CAT. CV-1 cells were transfected with MYH7-CAT along with various amounts of the expression plasmid for mouse TEAD-3 (B), mouse TEAD-1 (C), and mouse TEAD-4 (D). CAT activity for pCMV-CAT was taken as 100%. Data are expressed as the mean ± S.D. of at least three independent experiments. *, *P*<0.05; **, *P*<0.01; ***, *P*<0.001 of TEADs (−) vs. TEADs (+).

**Figure 2:**
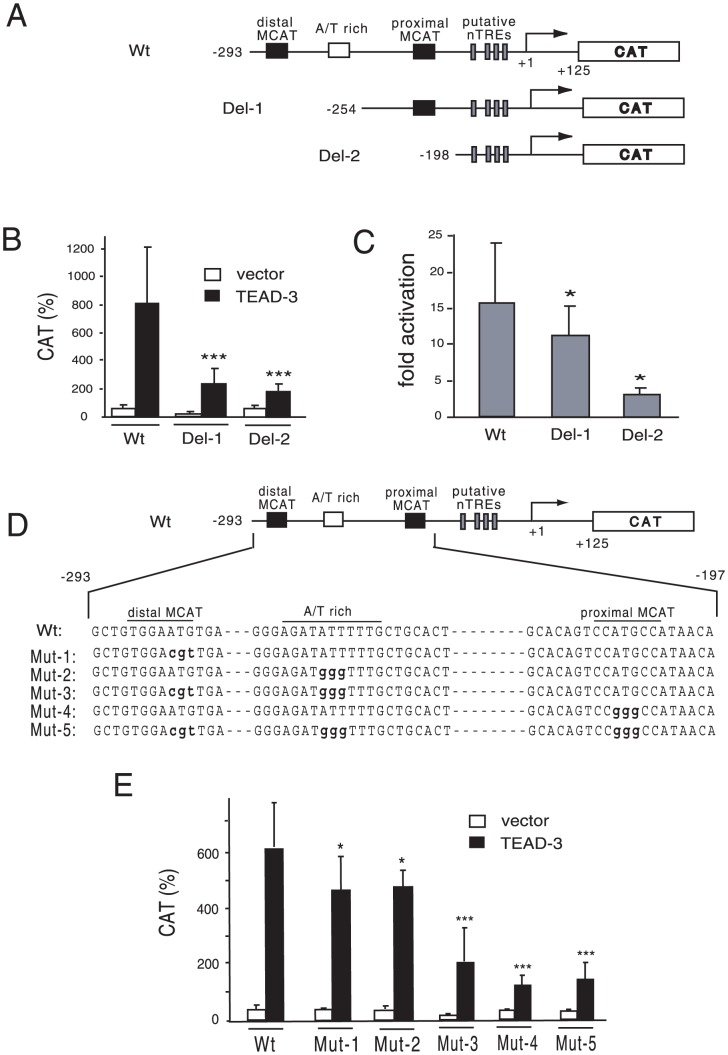
Two MCAT sites and an A/T-rich region are required for transactivation by TEAD-3. (A) A schematic representation of MYH7-CAT (wild-type) and its deletion constructs (Del-1 and Del-2-CAT). (B) CV-1 cells were transfected with MYH7 (Wt)-, Del-1 and Del-2-CAT along with the expression plasmid for mouse TEAD-3 (pXJ40-DTEF-1). Open bar, empty vector; solid bar, TEAD-3. CAT activity for pCMV-CAT was taken as 100%. Data are expressed as the mean ± S.D. of at least three independent experiments. ***, *P*<0.001 of the wild type (Wt) vs. mutants (Del-1 and 2). (C) The effects of deletions of the MCAT sites and an A/T-rich region are indicated as fold activation. CAT activity with TEAD-3 was divided by that without TEAD-3 to calculate fold activation. *, *P*<0.05 of the wild type (Wt) vs. deletion mutants (Del-1 and 2). The reduction in fold activation of Del-1 was modest because the magnitude of basal activity of this construct was lower than that of the wild-type (p = 0.078, Fig. 2B). (D) A schematic representation of MYH7-CAT (Wt) and its mutants (Mut1–5). Mutated nucleotides are indicated as lower case bold letters. (E) The effect of mutations of MCAT sites and the A/T-rich region. The experimental conditions were the same as (B). CAT activity for pCMV-CAT was taken as 100%. Data are expressed as the mean ± S.D. of at least three independent experiments. *, *P*<0.05; ***, *P*<0.001 of the wild type (Wt) vs. mutants (Mut-1 to 5).

**Figure 3:**
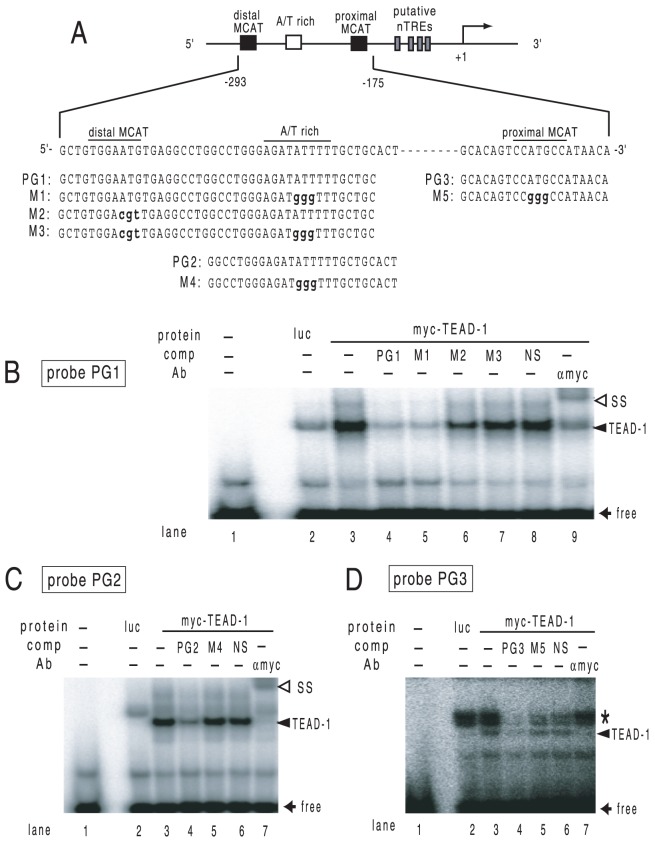
TEAD-1 recognizes MCAT sites and the A/T-rich region with various affinities. (A) Schematic of probe-PG1 (distal MCAT and the A/T-rich region), PG2 (the A/T-rich region) and PG3 (proximal MCAT). The mutated nucleotides of M1–5 are indicated as lower case bold letters. (B–D) Gel shift assay using radiolabeled PG1 (B), PG2 (C), and PG3 (D) with the *in vitro* translation of pcDNA3-6myc-TEAD-1. Solid arrowhead, TEAD-1 (*in vitro* translation of 6myc-TEAD-1); open arrowhead, supershift (SS) of TEAD-1 by the antibody against the myc peptide; solid arrow, free probe; luc, firefly luciferase (control). Asterisk indicates a non-specific band upstream to TEAD-1 (see text).

The structure of the human MYH7 promoter (nt −293/+125) is illustrated in Figure 1A. The DNA sequences of these elements are highly conserved among species. Because the firefly luciferase-based reporter gene may be artificially suppressed by T3/TR [16], [18], [26], we employed the CAT-based reporter system. On the basis of previous reports in which endogenous TEADs in rat car_diomyocytes may recognize these MCAT sites and the A/T-rich element [28], [30], [31], we transfected the expression plasmid for TEAD-3, TEAD-1, and TEAD-4 into CV-1 cells and examined their effect on MYH7 promoter activity. As shown in Figures 1B, C, and D, these TEADs potently activated the MYH7 promoter in a dose-dependent manner. We conducted deletion analyses of the MYH7 promoter (Fig. 2A) to confirm the importance of DNA recognition by TEADs. Although deletion of distal MCAT and the A/T-rich region (Del-1) decreased the magnitude of TEAD-3-induced activity (Fig. 2B), its basal activity was also slightly lower than that of the wild-type (p = 0.078), which resulted in a modest reduction in fold activation (Fig. 2C). The truncation of all MCATs and the A/T rich region (Del-2) strongly reduced TEAD-3-induced activity (Fig. 2B) as well as fold activation (Fig. 2C). We then performed mutation analysis (Fig. 2D). Although the reduction in promoter activity was modest for the distal MCAT (Mut-1) or A/T rich region (Mut-2) mutation, the mutation of both distal MCAT and the A/T rich region (Mut-3) as well as that of the proximal MCAT (Mut-4) markedly reduced MYH7 promoter activity (Fig. 2E). Similar results were obtained when all MCATs and the A/T rich region were mutated (Mut-5, Figs. 2D and 2E). Taken together, these results suggested that TEAD3-induced transactivation is mainly maintained by proximal MCAT and partially by distal MCAT and the A/T rich region. We performed a gel shift assay with ^32^P-radiolabeled oligo DNAs encompassing the distal MCAT site and an A/T-rich element (PG1), as well as the A/T-rich elements alone (PG2) (Fig. 3A). As shown in Figures 3B and 3C, binding signals were abolished or reduced by specific, but not non-specific competitors (Fig. 3B, lanes 4 and 8; Fig. 3C, lanes 4 and 6). The finding that competition of the binding signal by the mutant competitor, M1 (Fig. 3B, lane 5), was much clearer than that by M2 (lane 6) indicated that TEAD-1 binds the A/T-rich element with higher affinity than distal MCAT. Similar results were obtained when we used proximal MCAT (PG3) as a probe (Fig. 3D, lanes 4, 5, and 6). These bands were supershifted (Fig. 3B, lane 9; Fig. 3C, lane 7) or eliminated (Fig. 3D, lane 7) by the anti-myc antibody. In Figure 3D, we found a band that was abolished by specific competitors (asterisk, lane 3). This signal was thought to be nonspecific because it was observed in the control lane (lane 2) and was not affected by the anti-myc antibody (lane 7) although we could not exclude the possibility that the *in vitro* translation system may contain a TEAD family transcription factor other than TEAD-1. Collectively, TEAD family transcription factors bind with the two MCAT sites and the A/T-rich element with various affinities, resulting in MYH7 promoter activation.

### T3-bound TR (T3/TR) inhibites TEADs-induced activity of the MYH7 promoter

**Figure 4:**
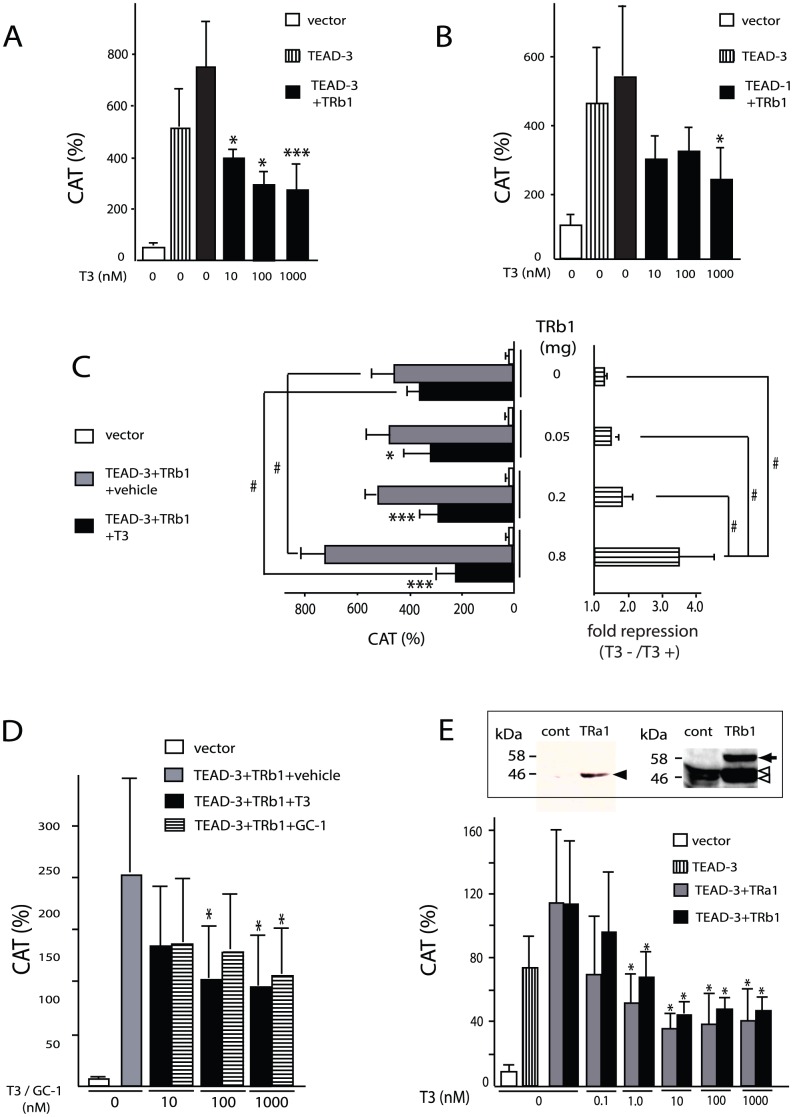
T3-bound TRs (T3/TRs) repress TEAD-induced activity of the MYH7 promoter. (A) and (B) T3/TRβ1 represses promoter activity of the MYH7 gene induced by TEAD-3 (A) or TEAD-1 (B). MYH7-CAT (wild-type) was transfected into CV-1 cells along with the expression plasmid for human TRβ1, mouse TEAD-3, or TEAD-1. *, *P*<0.05; ***, *P*<0.001 of T3 (−) vs. T3 (+). (C) Dose dependency of the amount of TRβ1 expressed. MYH7-CAT (2.0 µg) was co-transfected with the expression plasmid for human TRβ1 (0–0.8 µg) into CV-1 cells along with or without mouse TEAD-3 (0.2 µg) under the same conditions as those described in (A). In the left panel, CAT activities in the presence or absence of 1 µM T3 are indicated. The results are means ± S.D. for three independent experiments. *, *P*<0.05; ***, *P*<0.001 of T3 (−) vs. T3 (+). #, *P*<0.05. In the right panel, TRβ1 dose-dependency is indicated as fold repression. CAT activity without T3 was divided by that with 1 µM T3 to calculate fold activation. #, *P*<0.05. (D) Dose dependency of T3 or GC-1. MYH7-CAT, TEAD-3, and TRβ1 were expressed in CV-1 cells under the same conditions as those described in (A) and 0–1000 nM of T3 or GC-1 was supplemented. The results are means ± S.D. for three independent experiments. *, *P*<0.05 vs. TEAD-3 plus TRβ1 with vehicle. (E) TRα1 as well as TRβ1 inhibit the MYH7 promoter by T3. MYH7-CAT, TEAD-3, and FLAG-tagged TRα1 (gray bar) or TRβ1 (solid bar) were expressed in CV-1 cells under the same conditions as described in (A), and 0–1000 nM of T3 was supplemented. The expression of FLAG-tagged TRα1 and TRβ1 transfected into CV-1 cells were demonstrated by Western blot with antibody against FLAG and N-terminal region of TRβ1 [16], [26], [27], respectively (inset). Solid arrowhead, TRα1; solid arrow, TRβ1; open arrowheads, non-specific bands; cont, empty plasmid. The numbers on the left side of each panel indicate molecular mass markers (kDa). The results are means ± S.D. for three independent experiments. *, *P*<0.05 of T3 (−) vs. T3 (+). In these reporter assays (A–E), the expression level of pCMV–CAT was adjusted to a value of 100.

Because previous studies showed that TRβ1, but not TRα1, may mediate negative regulation of the MYH7 gene [4], [19], we initially employed TRβ1 to test the effect of T3 on TEAD-dependent transactivation of the MYH7 promoter. We found that transactivation of the MYH7 promoter by TEAD-3 was inhibited by 10 nM T3 in the presence of TRβ1 (Fig. 4A). TEAD-1-induced transactivation was also inhibited by T3-bound TRβ1 (T3/TRβ1) (Fig. 4B). The observation that TEAD-induced activity of the MYH7 promoter was repressed by T3/TRβ1 in non-cardiac CV-1 cells suggests that a cardiomyocyte-specific factor other than TEADs and TR is not essential for negative regulation of the MYH7 gene. As shown in Figure 1A, a constitutive inhibitory sequence (β negative regulatory element, βNRE) has been reported at nt −332/−300 [2], [31]. However, inhibition by T3/TRβ1 is independent of this sequence because our MYH7-CAT lacks it. As shown in the left panel of Figure 4C, when TEAD-3 was co-expressed (gray bar), MYH7 promoter activity was significantly increased by the large amount of unliganded TRβ1 (0.8 µg). The T3-dependent repression presented by the fold repression (right panel) was in a TRβ1 dose-dependent manner. As shown in the left panel, the MYH7 promoter was not activated by unliganded TRβ1 without TEAD-3 (open bar) at an amount sufficient for T3-dependent inhibition of the MYH7 promoter induced by TEAD-3 (gray and solid bars). Thus, unliganded TRβ1 *per se* is not the transcriptional activator for basal activity of the MYH7 promoter. The TRβ-selective T3 analog, GC-1 [14], also suppressed the promoter activity induced by TEAD-3 (Fig. 4D). Taking advantage of the fact that CV-1 cells do not express endogenous TR [16], we examined whether TRα1 may mediate negative regulation of the MYH7 gene by T3. Unexpectedly, not only TRβ1, but also TRα1 mediated the repressive effect of T3 on the MYH7 promoter (Fig. 4E). The expression for both receptors was confirmed by Western blot (inset). Taken together, TRα1 as well as TRβ1 inhibited TEAD-induced transactivation of the MYH7 promoter. Because the cardiac expression of TRα1 mRNA is two- to three-fold higher than that of TRβ1 [7], [8], we tested whether TRα1 may exhibit a dominant negative effect on the suppression induced by GC-1-bound TRβ1 (GC-1/TRβ1) [15], [42]. Although increasing amount of TRα1 (0 to 0.8 µg/dish) moderately relieved the inhibition of the MYH7 promoter by GC-1/TRβ1, similar de-repression by TRα1 was observed when this promoter was inhibited by T3/TRβ1 (data not shown). Thus, in this assay system, we could not conclude that TRα1 has the dominant negative effect on the negative regulation by GC-1/TRβ1.

### T3-dependent repression requires DBD of TRβ1

**Figure 5:**
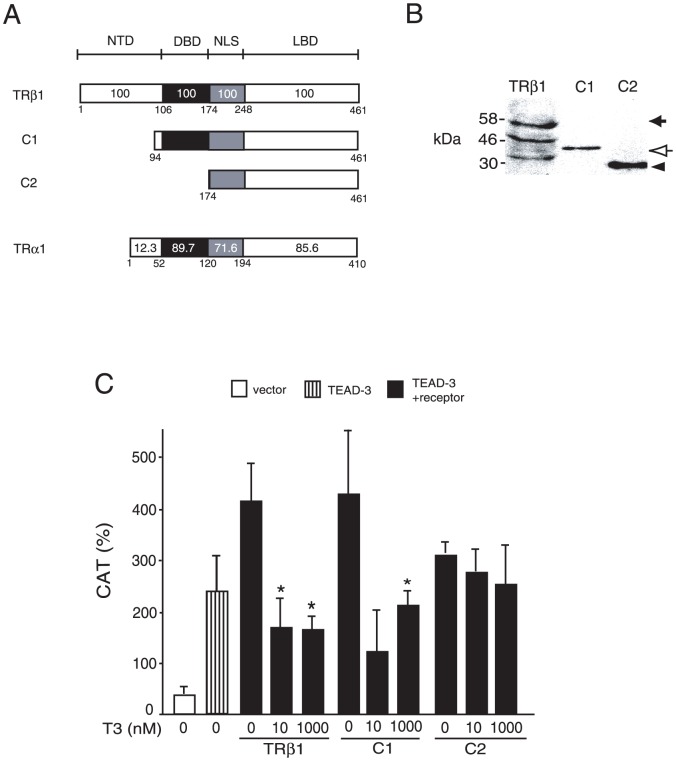
T3-dependent repression requires DBD. (A) Schematic representations of wild-type TRβ1, its mutants (C1 and C2), and wild-type TRα1. NTD, N-terminal domain; DNA, DBD; NLS, nuclear localization signal; T3, LBD. The numbers within and under the box represent amino acid homology (%) and codon numbers, respectively. (B) Expression of wild-type or mutant TRβ1s (C1 and C2) in CV1 cells. Whole cell extracts of CV1 cells transfected with equal amounts of expression plasmids for wild-type and mutant TRβ1s were analyzed by Western blotting with the anti-FLAG antibody. Solid arrow, wild-type TRβ1; open arrow, C1; solid arrowhead, C2. The numbers on the left side of each panel indicate molecular mass markers (kDa). (C) In the presence of T3, TEAD-3-induced activity of MYH7-CAT was inhibited by TRβ1 (wild-type) and C1, but not by C2. Under the same conditions as described in Figure 4A, CV-1 cells were transfected with 0.4 µg of the expression plasmid for human TRβ1 (wild-type), C1 or C2 in the presence or absence of T3. The results are means ± S.D. for six independent experiments. *, *P*<0.05 of T3 (−) vs. T3 (+).

The above findings demonstrated that both TRα1 and TRβ1 repress the MYH7 promoter activated by TEAD-3 upon T3-binding with their LBDs (Fig. 4E). Because the amino acid sequences of DBD and LBD in TRα1 have high homology with those in TRβ1 (Fig. 5A), we speculated that, in addition to LBD, DBD may also be involved in T3-dependent repression of the MYH7 gene. We aimed to examine the function of the truncation mutants, C1 and C2 (Fig. 5A), both of which preserved the amino acid sequence required for the nuclear localization signal (NLS). The expression levels of these mutants and wild-type TRβ1 are shown in Figure 5B [26], [27]. As predicted, full-length TRβ1 and C1 repressed TEAD-3-induced activation of the MYH7 promoter in the presence of T3 (Fig. 5C), whereas C2 did not. Hence, DBD are required for negative regulation of the MYH7 promoter by T3.

### Reported nTREs are not necessary for repression of the MYH7 promoter by T3/TR

**Figure 6:**
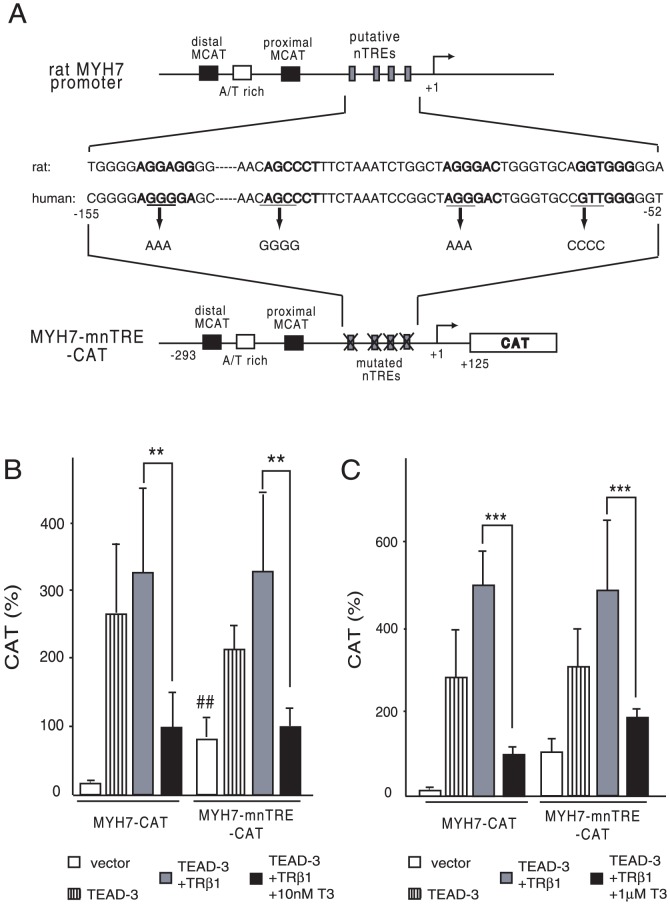
Reported nTREs are dispensable for repression of the MYH7 promoter by T3/TR. (A) Schematic representation of the rat MYH7 promoter and reported nTREs. Transcription start site is indicated as +1. The reported nTREs in the rat MYH7 gene (upper panel) and their corresponding sequences of the human MYH7 gene (middle panel) are indicated as upper case bold letters. In MYH7-mnTRE-CAT, the putative four nTREs corresponding to those in the rat MYH7 gene were mutated (lower panel). (B) and (C) Reported nTREs were not essential for repression of the MYH7 promoter by T3/TR. Under the same conditions as those described in Figure 4A, CV-1 cells were transfected with 2.0 µg of MYH7-CAT or MYH7-mnTRE-CAT in the presence or absence of 10 nM (B) or 1 µM T3 (C). CAT activity was measured under the same conditions as in Figure 4A. The expression level of pCMV–CAT was adjusted to a value of 100. The results are means ± S.D. for three independent experiments. **, *P*<0.01; ***, *P*<0.005 of T3 (−) vs. T3 (+). ##, P<0.01 of MYH7-CAT vs. MYH7-mnTRE-CAT.

The finding that DBD are required for T3-dependent inhibition (Fig. 5) raised the possibility that direct recognition of DNA by DBD may be necessary for the negative regulation of the MYH7 promoter by T3/TR. As shown in Figure 6A, four putative nTREs in rat MYH7 genes were suggested based on sequence homology with a single half-site (consensus sequence: AGGTCA) [21]. However, their functional relevance was not experimentally assessed. Because all of the sequences corresponding to rat nTREs were included in the human MYH7 promoter sequence in MYH7-CAT (Fig. 6A), we attempted to mutate these sequences to generate MYH7-mnTRE-CAT. As shown in Fig. 6B and 6C, the mutation of all of these nTREs significantly increased the TEAD3-independent activity (open bars) via unknown mechanism. However, the repression by TRβ1 in the presence of 10 nM and 1 µM T3 was clearly maintained, which suggested that nTREs are not crucial for inhibition by T3/TR.

### TR-DBD directly interacts with the TEA domain in a T3-independent manner

**Figure 7:**
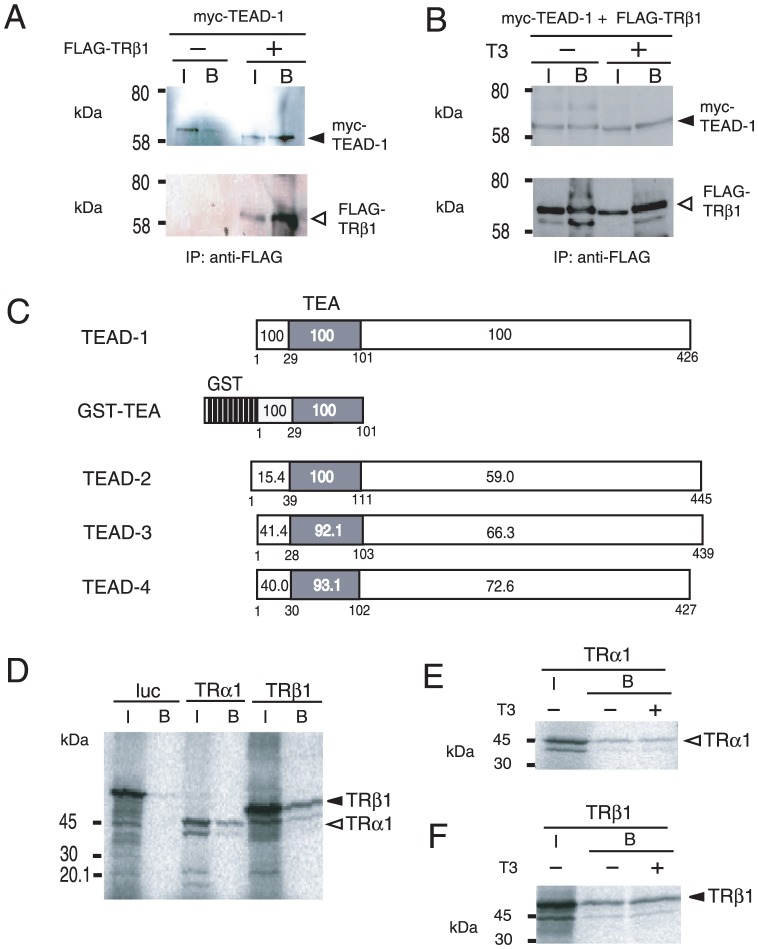
TRs directly interact with TEAD-1. (A) Co-immunoprecipitation of TRβ1 with TEAD-1. The expression plasmids for 6myc-TEAD-1 (pcDNA3-6myc-TEAD-1) were transfected into CV-1 cells with or without FLAG-TRβ1. Whole-cell extracts were immunoprecipitated with the anti-FLAG M2 affinity gel and analyzed by Western blotting with the anti-myc antibody. I, input; B, bound; IP, immunoprecipitation. The numbers on the left side of each panel indicate molecular mass markers (kDa). Solid and open arrowheads indicate 6myc-TEAD-1 (upper panel) and FLAG-TRβ1 (lower panel), respectively. (B) The interaction of TRβ1 with TEAD-1 was T3-independent. In the presence or absence of 1 µM T3, co-immunoprecipitation of TRβ1 with TEAD-1 was performed under the same conditions as in (A). (C) Schematic representations of TEAD-1, GST-TEA, and TEAD-2, 3, and 4. TEA, the TEA domain. The numbers within and under the box represent amino acid homology (%) and codon numbers, respectively. (D) Both TRα1 and TRβ1 interact directly with the TEA domain of TEAD-1. A GST pull-down assay was performed using GST-TEA and ^35^S-labeled *in vitro* translated TRα1, TRβ1, or firefly luciferase (luc). (E) and (F) The interaction of TEAD-1 with TRα1 (E) or TRβ1 (F) is T3-independent. A GST pull-down assay was performed in the presence or absence of 1 µM T3 under the same conditions as (D). The numbers on the left side of each panel indicate molecular mass markers (kDa). Solid and open arrowheads indicate TRα1 and TRβ1, respectively. I, input; B, bound.

**Figure 8:**
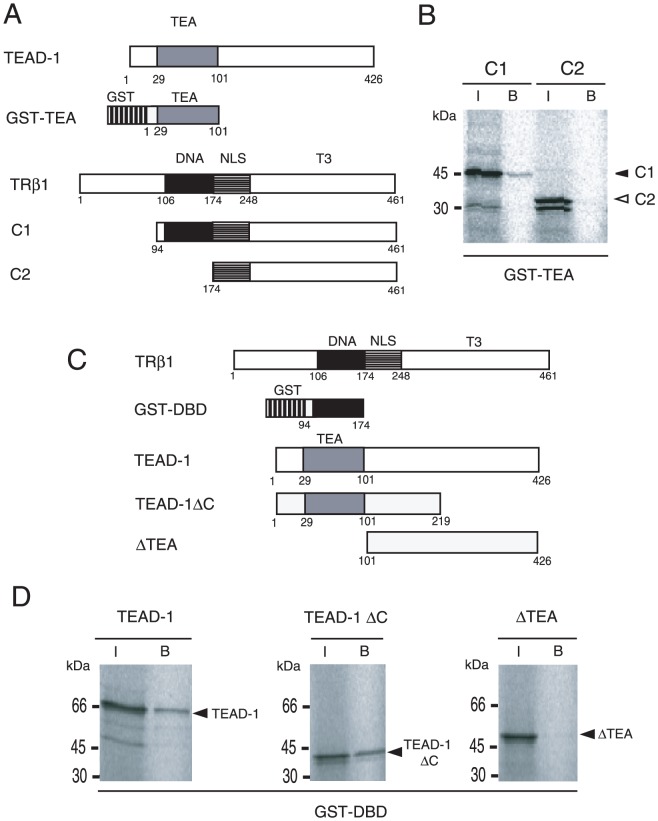
TR-DBD directly binds the TEA domain. (A) Schematic representations of wild-type TRβ1, its mutants (C1 and C2), and GST-TEA. The numbers under the box represent codons. DNA, DBD; NLS, nuclear localization signal; T3, LBD; TEA, TEA domain of TEAD-1. (B) The TEA domain interacts with TRβ1-DBD, but not LBD. A GST pull-down assay was performed using GST-TEA and ^35^S-labeled *in vitro* translated C1 and C2 under the same conditions as those described in Figures 7D, E, and F. Solid and open arrowheads indicate C1 and C2, respectively. (C) Schematic representations of wild-type TRβ1, GST-DBD, TEAD-1, TEAD-1ΔC, and ΔTEA. The numbers under the box represent codons. (D) TRβ1-DBD interacts with the TEA domain of TEAD-1. A GST pull-down assay was performed using GST-DBD and ^35^S-labeled *in vitro* translated TEAD-1, TEAD-1ΔC, and ΔTEA under the same conditions as those described in (B). Solid arrowheads indicate TEAD-1 (left panel), TEAD-1ΔC (middle panel), and ΔTEA (right panel). I, input; B, bound.

It should be emphasized that the function of NHR-DBD is not limited to the direct recognition of DNA, but can also be associated with DNA-binding transcription factors *via* protein-protein interactions and modulate their transactivation function in a ligand-dependent manner (tethering mechanism) [16], [18], [43]–[45]. Based on the hypothesis that ligand-dependent inhibition can be detected only when T3/TR attenuates the activity of the DNA-binding transcription factor maintaining global transcription levels [16], we tested whether TRβ1 interacts with TEADs. As shown in Figure 7A, N-terminal six tandem myc-tagged mouse TEAD-1 (6myc-TEAD-1) was co-transfected with FLAG-tagged TRβ1 (FLAG-TRβ1) into CV-1 cells. We found that 6myc-TEAD-1 was co-immunoprecipitated with FLAG-TRβ1 (Fig. 7A), and this interaction was T3-independent (Fig. 7B). As shown in Figure 7C, the large part of the N-terminal amino acid sequence, i.e., TEA domains, is highly conserved among TEADs, whereas that of the remaining C-terminal regions has lower homology [32], [33]. Given that suppression by T3/TRβ1 is detected in transactivation of the MYH7 promoter by TEAD-1 as well as TEAD-3 (Figs. 4A and 4B), the TEA domain is thought to be the common interface of interaction with TRs. Indeed, glutathione S-transferase (GST) fused to the TEA domain (GST-TEA, Fig. 7C) interacted with ^32^S-labeled TRα1 and TRβ1 (Fig. 7D), suggesting the direct binding of TEAD-1 with TRs. As demonstrated using the co-immunoprecipitation assay (Fig. 7A), the association of GST-TEA with TRα1 and TRβ1 was again T3-independent (Figs. 7E and 7F). We then tested the binding of GST-TEA with the truncation mutants of TRβ1 (Fig. 8A). We revealed that DBD, but not the N-terminal domain of TRβ1, is necessary for the interaction with GST-TEA (Fig. 8B). Although both DBD and LBD have the amino acid sequences highly conserved between TRα1 and TRβ1 (Fig. 5A), LBD failed to interact with the TEA domain (Figs. 8A and 8B). With this observation in mind, we fused TRβ1-DBD with GST to generate GST-DBD (Fig. 8C). As shown in Figure 8D, GST-DBD bound with TEAD-1 (left panel). Although the interaction was not affected by truncation of the C-terminal domain (middle panel), it was abolished by deletion of the TEA domain (right panel), which confirmed that the TEA domain of TEAD-1 is required for the interaction with TRβ1-DBD. These findings coupled with the results shown in Figure 6 indicate that TR-DBD is not used for the direct recognition of putative nTREs in negative regulation of the MYH7 gene [2], [21], but is involved in the protein-protein interaction with the TEA domain of TEAD-1, which is highly conserved among TEADs (Fig. 7C).

### T3/TR represses TEAD-induced activity of the MYH7 promoter in the cultured cardiomyocytes, H9c2

**Figure 9:**
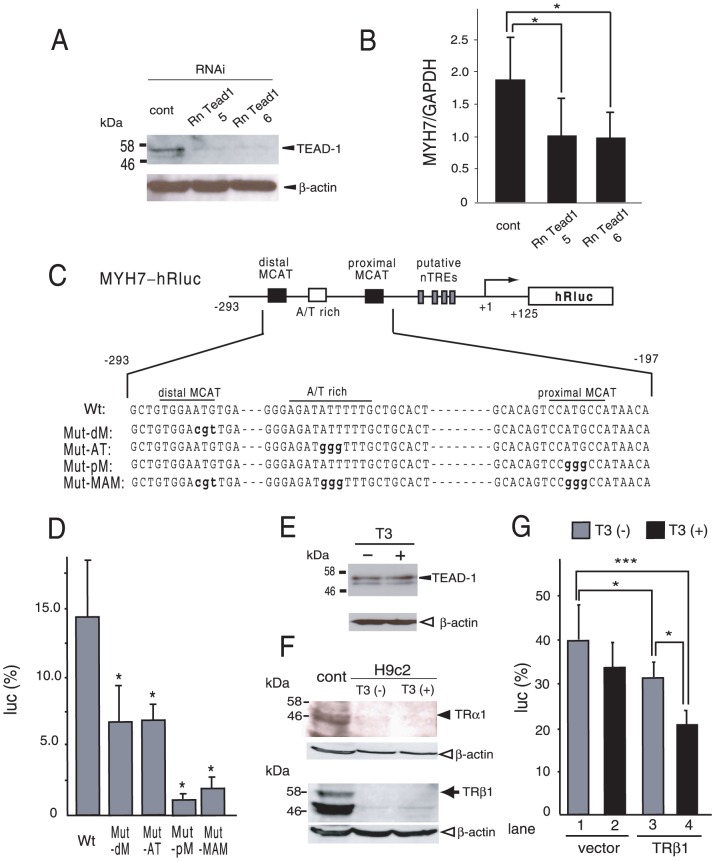
Basal expression of the MYH7 gene depends on TEADs and is inhibited by T3/TR in the rat embryonic cardiomyocytes, H9c2. (A) Western blots with antibodies against anti-TEAD-1 and β-actin show the effect of transfection of two sets of RNAi on TEAD-1 (Rn Tead1 5 or Rn Tead1 6) in H9c2. The position of TEAD-1 and β-actin are indicated as arrowheads. (B) Expression levels of the MYH7 gene measured by RT-PCR were reduced by siRNAs against TEAD-1. H9c2 cells incubated with siRNAs against TEAD-1 (Rn Tead1 5 or Rn Tead1 6) or control (cont) for 24 h. (C) A schematic representation of MYH7-hRluc (Wt) and its mutants (Mut-dM, AT, pM, and MAM). Mutated nucleotides are indicated as lower case bold letters. (D) Two MCAT sites and an A/T-rich region were necesary for basal activity of the MYH7 promoter. MYH7-hRluc (Wt) and its mutants (Fig. 9C) were transfected into H9c2 cells using Lipofectamine 2000. *, *P*<0.05 vs. MYH7-hRluc (Wt). The results are means ± S.D. for three independent experiments. (E) Expression levels of TEAD-1 are not affected by T3. The procedure for Western blotting is the same as (A). (F) Expression of TRs is not detected in H9c2 cells. Whole cell extracts of H9c2 cells were analyzed by Western blotting with the antibody against TRα1 (upper panel) and TRβ1 (lower panel). In the same gels, whole cell extracts of CV1 cells transfected with expression plasmids for TRα1 and TRβ1 were also analyzed by Western blotting with corresponding antibodies. Solid arrowhead, TRα1; solid arrow, TRβ1; open arrowhead, β-actin. (G) T3/TRβ1 inhibits MYH7 promoter activity in H9c2 cells. In the presence or absence of 1 µM T3, MYH7-hRluc (2.0 µg) with or without the expression plasmid for TRβ1 (0.2 µg) was transfected into H9c2 cells. The experimental procedure is the same as (D). The results are means ± S.D. for three independent experiments. *, *P*<0.05; ***, *P*<0.005. In reporter assays (D and G), the expression level of pGL4.74[hRLuc/TK] was adjusted to a value of 100.

To validate the importance of TEADs for basal activity of the MYH7 promoter, we performed RNA interference against endogenous TEAD-1 in H9c2 cells, which are derived from the rat embryonic heart [40]. When endogenous TEAD-1 expression was efficiently knocked down (Fig. 9A), MYH7 expression measured by RT-PCR was significantly reduced (Fig. 9B), suggesting the importance of TEAD-1 for global promoter activity of the MYH7 gene in H9c2 cells. To evaluate the possible involvement of other TEADs, we examined the effect of mutations of two MCAT sites and the A/T-rich element. Because of the low transfection efficiency of this cell line, MYH7-hRluc (Fig. 9C) was newly generated by fusing the MYH7 promoter (nt −293/+125) with modified *Renilla* luciferase (hRluc). As shown in Figure 9D, mutating proximal MCAT (Mut-pM) eliminated MYH7 promoter activity, whereas mutating the distal MCAT site (Mut-dM) or A/T rich element (Mut-AT) modestly reduced it. The reduction in activity by these mutations (Fig. 9D) was greater than the RNA knockdown of TEAD-1 (Fig. 9B), which suggests the partial involvement of TEADs other than TEAD-1 in transactivation of the MYH7 gene in H9c2 cells. Because it was previously confirmed that the DNA sequence of the hRluc coding region did not mediate artificial negative regulation by T3 [18], we attempted to examine the effect of T3/TR on MYH7 promoter activity. Endogenous TEAD-1 protein levels were not influenced by the 1 µM T3 treatment (Fig. 9E). Expression of endogenous TRs in H9c2 cells was not detected by Western blotting (Fig. 9F) and the effect of T3 (1 µM) on the MYH7 promoter was minimal (Fig. 9G, lanes 1 and 2). Overexpression of TRβ1 significantly (*p*<0.05) reduced MYH7 promoter activity (lanes 1 and 3), which may have been because of the small amount of endogenous T3 produced by H9c2 cells [46]. Importantly, the addition of 1 µM T3 significantly suppressed transcriptional activity of the MYH7 promoter (lanes 3 and 4), suggesting that, in cardiomyocytes, H9c2, T3/TR inhibits activity of the MYH7 gene, which is maintained by TEADs.

## Discussion

We established a reconstitution system with CV1 cells to investigate the involvement of TEADs in negative regulation of the MYH7 gene by T3/TR (Figs. 1–4). Although the results in Figures 2 and 3 suggest the importance of proximal MCAT, previous reports using endogenous TEADs [29], [30] and the results with H9c2 (Fig. 9D) indicated that distal MCAT and the A/T-rich region also play some roles in activating the MYH7 gene. In any case, our results support TEADs being a critical transcriptional activator for the MYH7 gene [1], [2], [28]–[32]. Using this system, we demonstrated that T3/TR inhibits human MYH7 promoter activity induced by TEADs (Fig. 4). Although previous studies have suggested that TRβ1, but not TRα1, may mediate negative regulation of the MYH7 gene by T3 [8], [19], our reconstitution system with CV-1 cells revealed that TEAD-dependent activation of the MYH7 promoter is also repressed by liganded TRα1 (Fig. 4E), which is a major TR in cardiac tissue [4], [7], [14]. This is supported by inhibition of MYH7 expression by T3 remained in TRβ1-deficient mice [20]. We also demonstrated that T3-dependent repression requires TRβ1-DBD (Fig. 5C), the amino acid sequence of which have high homology with that of TRα1 (Fig. 5A). Although we have shown here that 10 nM T3 inhibits the MYH7 gene via TRs in CV1 cells transfected with TEAD-3 (Fig. 4A), it should noted that the inhibition of this gene by T3 in vivo may be more potent than that observed in our reconstitution system [20]. In future, further study will be required.

In the presence of TRβ1, TEAD-induced activity of the MYH7 promoter was repressed by GC-1, a TRβ-selective T3 analog [14] (Fig. 4D). Importantly, TRβ-selective T3 analogs are expected to have a minimal effect on heart rate [14], [47], because heart rate is mainly regulated by TRα1, but not TRβ1 [14]. Moreover, TRβ-selective T3 analogs exhibited milder oxygen consumption than that of T3 [48], and liganded TRβ1 was shown to play a critical role in forming capillary networks in coronary endothelial cells of the pathologically hypertrophied heart [49]. Thus, TRβ-selective T3 analogs may be applicable for the treatment of heart failure by reducing MYH7 expression [1], [2], [38]. Although TRα1 mRNA is expressed two- to three-fold higher than that of TRβ1 in the cardiac tissues [7], [8], we could not detect the dominant negative effect by TRα1 on the inhibition by GC-1/TRβ1 (data not shown). The weak affinity of GC-1 with TRα1 [10-fold lower affinity than that with TRβ1 [14] may relieve the dominant negative effect by TRα1 that does not bind T3.

In contrast to rodents, reports on cardiac MYH7 expression in human subjects with hypo- and hyperthyroidism are limited. In large vertebrates including primates or rabbits [2], [7], MYH7 represents 70 to 90% of the total myosin heavy chain protein with the remainder being MYH6, whereas MYH7 accounts for only approximately 10% of the total myosin heavy chain protein in the cardiac tissue of adult rodents [8], [21]. Therefore, it may be difficult to detect increased MYH7 expression in human subjects with hypothyroid conditions [50]. In contrast, Kawana et al. [51] reported the findings of a myocardial biopsy from a subject with hyperthyroidism. Their immunohistochemical study suggested an increase in the percentage of MYH6 during the course of thyrotoxicosis returning to normal levels following treatment. Their findings may imply that, in human, T3 increased and decreased the expression of MYH6 and MYH7, respectively. In the present study, we demonstrated that T3/TR inhibited transactivation of the human MYH7 promoter by TEADs (Fig. 4). Consistent with these findings, the administration of exogenous T3 or T4 significantly reduced MYH7 expression in large animals, including the baboon [1], [52], rabbit [53], and calf [1]. In contrast, the contents of cardiac myosin isoforms were not altered in a case of sudden death of a female subject with hyperthyroidism [54]. Nevertheless, autopsy studies have indicated the existence of chronic congestive heart failure with endocardial fibrosis. Because both pressure overload [4] and cardiac fibrosis [55] are known to increase MYH7 expression, the repressive effect by T3 might be masked in this case.

**Figure 10:**
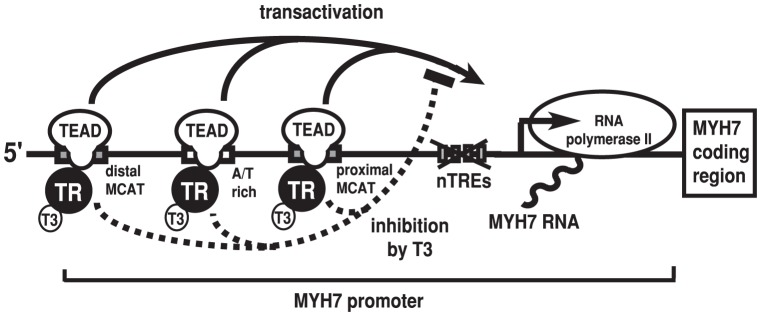
Tethering model of T3-dependent negative regulation of the MYH7 gene. T3/TR interferes with the transactivation function of TEADs. In this model, putative nTREs are not necessary for inhibition by T3/TR. TR-DBD is used for the protein-protein interaction with the TEA domain of TEADs, but not for direct recognition of the DNA sequence.

Although we cannot exclude the involvement of the far upstream DNA sequence in the MYH7 gene (Fig. 1A), the results in Figure 4 clearly indicate that the DNA sequence, nt −293/+125, functions to mediate T3-dependent inhibition in CV-1 cells as long as TR and TEADs are co-expressed. Regarding negative regulation of the TSHβ gene by T3, we previously proposed the tethering model in which TR interferes with the transactivating function of GATA2 in a T3-dependent manner [16], [27]. The following findings of T3-induced negative regulation of the MYH7 gene are reminiscent of that in the TSHβ gene, although the activator for the MYH7 gene (i.e., TEADs) is distinct from that of the TSHβ gene (i.e., GATA2). First, T3-induced inhibition of the MYH7 promoter is readily detected even in CV-1 cells co-expressed with TR and TEADs (Fig. 4), which suggests that negative regulation of this gene may not require tissue-specific factors other than TRs and TEADs. Second, although the intact DBD of TR is required for the inhibition of TEAD-induced activation by T3/TR (Fig. 5), single half site-like sequences reported as putative nTREs [2], [21] are dispensable for MYH7 gene inhibition by T3 (Fig. 6). In this regard, the function of so-called nTREs [16] proposed based on sequence similarity with the half site should be tested [45]. Moreover, the hypothesis that these nTREs play a role in MYH7 gene repression by a microRNA, miR208a [56], should be reconsidered. Third, although MYH7 expression was shown to be increased with hypothyroidism [6], [7], [20], unliganded TR *per se* is not a transcriptional activator (Fig. 4C). Indeed, the results shown in Figures 1, 2, and 9 and the findings of previous studies [1], [2], [28]–[32] clearly suggest that TEADs are the main transcriptional activators for the MYH7 gene. The results in Figure 4 indicate that TEAD-induced transcriptional activation of the MYH7 promoter (nt −293/+125) is required to detect negative regulation by T3/TR. Finally, we found that TR-DBD directly interacts with the TEA domain (Fig. 8), which is highly conserved among TEADs (Fig. 7). Although the central domain of TR (C-domain) has been designated as DBD because it directly recognizes DNA sequences of TREs in positive regulation [11], it also functions as the interface for an association with DNA-binding transcription factors *via* a protein-protein interaction [16], [27], [43]–[45]. The abovementioned results (Figs. 7 and 8) coupled with the finding that nTREs were dispensable for T3-dependent inhibition (Fig. 6) suggest that TR-DBD is not used for the direct recognition of nTRE, but for a protein-protein interaction with the TEA domain of TEADs (Fig. 7C). This interaction may also account for the observation that unliganded TRβ1 functions as a weak co-activator for TEAD-3 (Fig. 4C, gray bar in the left panel). Taken together, we propose that the negative regulation of MYH7 is mediated *via* a tethering mechanism in which liganded TR attenuates the transactivation function of TEADs *via* a protein-protein interaction (Fig. 10) as in the case of GATA2 in negative regulation of the TSHβ gene [16], [27].

In addition to our tethering model (Fig. 10) or nTRE hypothesis [2], [21], three other possibilities exist for negative regulation of the MYH7 gene by T3/TR. First, MYH7 expression may be upregulated by the microRNA, miRNA-27a, *via* inhibition of TRβ1 expression [19]. This stems from the hypothesis that TRβ1, but not TRα1, may mediate negative regulation of the MYH7 gene [4]. However, this is not the case (Fig. 4E) [20]. Second, because the sequence similar to the GATA-responsive element exists in the A/T-rich region [57], GATA-induced activation may mediate inhibition by T3/TR, as is the case of the TSHβ gene [16], [27]. Nevertheless, an *in vitro* study revealed that GATA family transcription factors did not directly bind with this region [57] and that GATA4 alone did not activate the MYH7 promoter [58]. Finally, antisense RNA against MYH7 heterogeneous nuclear RNA (hnRNA) may be increased by T3/TR *via* positive TREs of the MYH6 promoter, which is located 4.0 kb downstream of the MYH7 gene in rat chromosome 15 [59], [60]. However, this hypothesis does not account for the following observations. i) Negative regulation by T3/TR is clearly observed in the reporter genes fused to the MYH7 promoter (Fig. 4) [15], [21], [61]. Because these reporter constructs lacked the sequence corresponding to MYH7 hnRNA, its transcriptional repression by T3 should be independent of antisense RNA against MYH7 hnRNA. ii) An inverse relationship between MYH7 and MYH6 mRNA levels has not been observed in mice deficient for TRs [20].

Given that T3-dependent negative regulation of the MYH7 gene is mediated by interactions between TRs and TEADs (Fig. 10), what is the final target of T3 signaling? Recent analyses with chromatin immunoprecipitation assays have suggested that histone modification is involved in negative regulation of the MYH7 promoter [62], [63]. One plausible explanation is that T3-induced transcriptional repression may be mediated by NCoR or SMRT associated with histone deacetylases {[16] and references therein}. However, T3-dependent inhibition of the MYH7 gene was maintained in knock-in mice with the NCoR mutant, which does not interact with unliganded TR {[22] and references therein}. Likewise, no histological abnormalities except for the lung were reported in knock-in mice with the SMRT mutant, which does not associate with unliganded TR [64]. Of note, nucleosome densities at the transcriptionally active promoters are often low [65]. This may imply that there are relatively few histones around the transcription start site of the MYH7 gene (Fig. 10) immediately before inhibition by T3. From this point of view, T3-induced transition from transcriptional activation to repression may be controlled by a mechanism other than histone modification. Mediator complex is known to directly bind with RNA polymerase II and regulate its enzymatic activity positively or negatively [17]. MED1, a constituent of Mediator complex, not only interacts with T3/TR *via* its LXXLL motifs, but also functions as a co-activator for GATA2 [27]. Based on this finding, we previously suggested that MED1 may play a role in negative regulation of the TSHβ gene by T3 [16], [27]. Interestingly, MED1 may also play a role in transcriptional regulation of the MYH7 gene because MYH7 expression was increased in mice in which the MED1 gene was deleted specifically in the cardiomyocytes and skeletal muscle [66]. However, further studies are necessary because TSHβ expression decreased, rather than increased in mice heterozygous for the MED1 gene {[16] and reference therein}.

Microarray analyses have indicated that approximately 40% of cardiac genes are repressed by T3 [67], [68]. For example, T3 was shown to inhibit the expression of angiotensin II type 1 receptor [69] and that of phospholamban, an inhibitor of the sarcoplasmic reticulum calcium ATPase 2a protein [7]. Angiotensin II type 1 receptor is a key component of the pathogenesis of heart failure, and gene inactivation of phospholamban has been shown to enhance basal cardiac contractility. In this study, we investigated the mechanism of T3/TR-induced negative regulation of the MYH7 gene, the expression of which is a molecular marker for heart failure [1], [2], [38]. Interestingly, both TEAD-3 and GATA2 are targets of the protein kinase C pathway from membrane receptors. The α1-adrenaline pathway has been shown to stimulate MYH7 expression [2], [32], [37] presumably *via* TEAD-3 [70], while the signal from thyrotropin-releasing hormone potentiates GATA2 transcriptional activity [16], [71]. Therefore, MCAT sites and the A/T-rich element in the MYH7 gene (Fig. 10) and GATA-responsive elements in the TSHβ gene appear to be points of convergence for both activating and repressing signals. Given that ligand-dependent inhibition is detectable only when liganded NHR attenuates transactivation by the transcription factor essential for overall transcription, identification of such an activator may provide a clue to explore the mechanism of ligand-dependent negative regulation [16], [18], [26].
