# Correction: Essential Role of TEA Domain Transcription Factors in the Negative Regulation of the MYH 7 Gene by Thyroid Hormone and Its Receptors

**DOI:** 10.1371/journal.pone.0106385

**Published:** 2014-08-18

**Authors:** The PLOS ONE Staff

The image for Figure 4 is incorrect. Please see the correct Figure 4 here.

**Figure 4:**
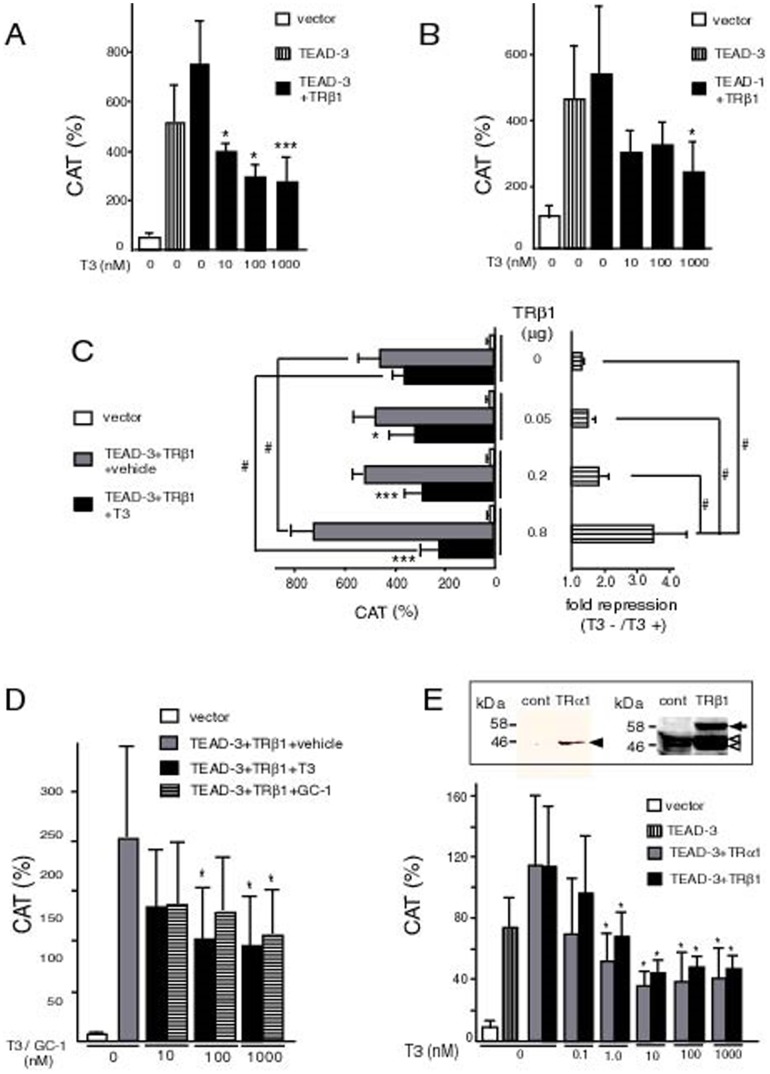
T3-bound TRs (T3/TRs) repress TEAD-induced activity of the MYH7 promoter. (A) and (B) T3/TRβ1 represses promoter activity of the MYH7 gene induced by TEAD-3 (A) or TEAD-1 (B). MYH7-CAT (wild-type) was transfected into CV-1 cells along with the expression plasmid for human TRβ1, mouse TEAD-3, or TEAD-1. *, P<0.05; ***, P<0.001 of T3 (−) vs. T3 (+). (C) Dose dependency of the amount of TRβ1 expressed. MYH7-CAT (2.0 µg) was co-transfected with the expression plasmid for human TRβ1 (0–0.8 µg) into CV-1 cells along with or without mouse TEAD-3 (0.2 µg) under the same conditions as those described in (A). In the left panel, CAT activities in the presence or absence of 1 µM T3 are indicated. The results are means ± S.D. for three independent experiments. *, P<0.05; ***, P<0.001 of T3 (−) vs. T3 (+). #, P<0.05. In the right panel, TRβ1 dose-dependency is indicated as fold repression. CAT activity without T3 was divided by that with 1 µM T3 to calculate fold activation. #, P<0.05. (D) Dose dependency of T3 or GC-1. MYH7-CAT, TEAD-3, and TRβ1 were expressed in CV-1 cells under the same conditions as those described in (A) and 0–1000 nM of T3 or GC-1 was supplemented. The results are means ± S.D. for three independent experiments. *, P<0.05 vs. TEAD-3 plus TRβ1 with vehicle. (E) TRα1 as well as TRβ1 inhibit the MYH7 promoter by T3. MYH7-CAT, TEAD-3, and FLAG-tagged TRα1 (gray bar) or TRβ1 (solid bar) were expressed in CV-1 cells under the same conditions as described in (A), and 0–1000 nM of T3 was supplemented. The expression of FLAG-tagged TRα1 and TRβ1 transfected into CV-1 cells were demonstrated by Western blot with antibody against FLAG and N-terminal region of TRβ1 [16], [26], [27], respectively (inset). Solid arrowhead, TRα1; solid arrow, TRβ1; open arrowheads, non-specific bands; cont, empty plasmid. The numbers on the left side of each panel indicate molecular mass markers (kDa). The results are means ± S.D. for three independent experiments. *, P<0.05 of T3 (−) vs. T3 (+). In these reporter assays (A–E), the expression level of pCMV–CAT was adjusted to a value of 100.
